# Roles of tRNA metabolism in aging and lifespan

**DOI:** 10.1038/s41419-021-03838-x

**Published:** 2021-05-26

**Authors:** Zheng Zhou, Bao Sun, Dongsheng Yu, Meng Bian

**Affiliations:** 1 Department of Chinese Medicine, The First Affiliated Hospital of Zhengzhou University, Zhengzhou, 450000 China; 2 Department of Pharmacy, The Second Xiangya Hospital, Central South University, Changsha, 410011 China; 3 Institution of Clinical Pharmacy, Central South University, Changsha, 410011 China

**Keywords:** Ageing, Genetics research

## Abstract

Transfer RNAs (tRNAs) mainly function as adapter molecules that decode messenger RNAs (mRNAs) during protein translation by delivering amino acids to the ribosome. Traditionally, tRNAs are considered as housekeepers without additional functions. Nevertheless, it has become apparent from biological research that tRNAs are involved in various physiological and pathological processes. Aging is a form of gradual decline in physiological function that ultimately leads to increased vulnerability to multiple chronic diseases and death. Interestingly, tRNA metabolism is closely associated with aging and lifespan. In this review, we summarize the emerging roles of tRNA-associated metabolism, such as tRNA transcription, tRNA molecules, tRNA modifications, tRNA aminoacylation, and tRNA derivatives, in aging and lifespan, aiming to provide new ideas for developing therapeutics and ultimately extending lifespan in humans.

## Facts

1. tRNAs are important participants in protein translation and are involved in various physiological and pathological processes.
2. tRNA-associated metabolism is closely associated with aging and lifespan.
3. The enzymes related to tRNA metabolism could be potential targets for future therapeutic interventions in aging and lifespan.

## Open questions

1. Is tRNA metabolism involved in the regulation of aging and lifespan mainly by affecting protein synthesis?
2. What is the molecular mechanism by which tRNA derivatives regulate aging and lifespan?
3. Is there potential for practical clinical applications based on findings concerning tRNAs in the context of aging and lifespan?

## Introduction

Transfer RNAs (tRNAs) are important participants in protein translation, which transport their cognate amino acids to the ribosome. There are more than 600 putative tRNA genes in the human genome, some of which are transcribed into precursor tRNAs (pre-tRNAs) by RNA polymerase III (Pol III)^1^. Subsequently, pre-tRNAs are transformed into mature tRNAs after a series of processing and modification processes, which are characterized by a “clover” secondary structure as well as an L-shaped tertiary structure^2^. After maturation, tRNAs are charged with their cognate amino acids through the aminoacylation reactions mediated by aminoacyl-tRNA synthetases (ARSs), thereby participating in protein translation^3^. Of note, tRNAs will be cleaved into fragments with regulatory functions under stress conditions^4–6^. In general, normal tRNA metabolism is essential to maintain the stability and functions of tRNA molecules, while the defects in certain tRNA biogenesis proteins cause various human diseases, including cancer, neurological disorders, immunodeficiency, and diabetes mellitus^7–10^.

**Fig. 1:**
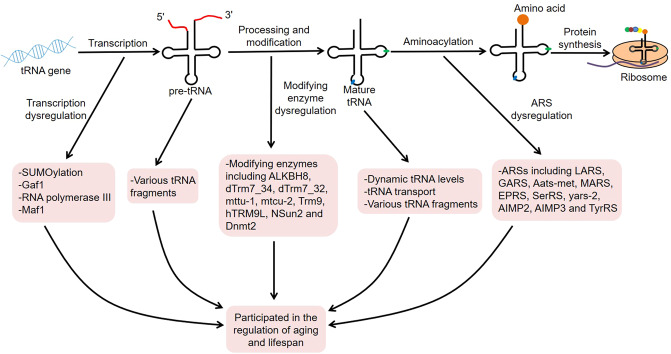
Roles of tRNA-associated metabolism in aging and lifespan. The tRNA genes are transcribed into pre-tRNAs, which are then transformed into mature tRNAs after a series of processing and modifications. Subsequently, mature tRNAs are involved in protein translation. During this process, tRNA-associated metabolism, such as tRNA transcription, tRNA molecules, tRNA modifications, tRNA aminoacylation, and tRNA derivatives, plays an important role in aging and lifespan.

Aging is a complex physiological process, usually manifested by a gradual decline in organ function, as well as an increase in disease incidence and risk of death. It is reported that the global population over 65 will reach 1.6 billion by 2050^11^. In fact, delaying biological aging or extending healthspan is an eternal theme of human health^12,13^. Strikingly, tRNAs play an important role in aging and lifespan. For example, the serum levels of mitochondrial tRNAs and ribosomal RNAs (rRNAs) will increase with age, which may be related to mitochondrial dysfunction during the aging process^14^. Another research discovered that Pol III was a downstream molecule of Target of Rapamycin Complex 1 (TORC1), and its inhibition could extend organismal lifespan^15^. Moreover, the deletion of nucleoporin Nup100 could regulate the life span of *Saccharomyces cerevisiae* by inhibiting the nuclear export of specific tRNAs^16^. Therefore, tRNAs are closely related to aging biology and thus participate in the regulation of age-related diseases and lifespan. Here, we focus on the roles of tRNA-associated metabolism, such as tRNA transcription, tRNA molecules, tRNA modifications, tRNA aminoacylation, and tRNA derivatives, in aging and lifespan, which may serve as novel targets for lifespan extension (Fig. 1).

## Roles of tRNA metabolism in aging and lifespan

### tRNA transcription in aging and lifespan

Small ubiquitin-related modifier modification (SUMOylation) is a highly dynamic post-translational modification that has been confirmed to be related to transcriptional repression^17,18^. Meanwhile, many studies have linked SUMOylation to aging process^19,20^. In eukaryotes, three essential RNA polymerases are evolutionarily conserved enzymes responsible for the transcription of their nuclear genomes. Of these, Pol I mainly transcribes the 25S rRNA precursor, Pol II transcribes various messenger RNAs (mRNAs), while Pol III transcribes short RNAs such as tRNAs and 5S rRNA^21,22^. Interestingly, SUMO machinery was widely distributed in the genome, especially at the promoters of histone and protein biogenesis genes, as well as Pol I-transcribed rRNA genes and Pol III-transcribed tRNA genes^23^. Surprisingly, the SUMO machinery was selectively retained on histone and tRNA genes and released in large quantities from other chromatin in senescent cells, indicating that maintaining the suppression of histone and tRNA loci was beneficial to the stability of the aging state^23^. These data support that SUMOylation-mediated coordinated repression of a transcriptional program is associated with cell growth and proliferation.

It is well-known that TORC1 is an important longevity determinant among many species^24–26^. Recent studies observed that the GATA transcription factor Gaf1 deficiency could shorten the normal chronological lifespan and reduce the lifespan extension caused by TORC1 inhibition in yeast^27^. Specifically, upon TORC1 block, Gaf1 served as a transcription factor downstream of TORC1 that directly bound to Pol III-transcribed tRNA genes and inhibited their transcription, thereby promoting longevity by inhibiting translation. Strikingly, Pol III mediated the longevity-promoting effects of TORC1 inhibition^15^. In this condition, systemic Pol III deficiency could facilitate the longevity in yeast, flies, and worms, and gut-specific inhibition of Pol III in adult worms or flies was sufficient to prolong the lifespan, which might be related to the reduced protein synthesis and increased resistance to proteotoxic stress. Importantly, the effects of Pol III inhibition and rapamycin treatment on lifespan extension were not additive^15^. Rapamycin treatment suppressed the phosphorylation of TORC1 substrate in the intestine, while the gut-specific Pol III inhibition did not, indicating that Pol III acted as a downstream molecule of TORC1 to regulate lifespan.

TORC1 directly phosphorylated MAF1 homolog negative regulator of Poll III (Maf1) at multiple sites and thus controlled its localization and Pol III-mediated transcription^28–30^. Notably, inhibition of TORC1 by rapamycin treatment reduced pre-tRNA levels in whole flies, and overexpression of gut-specific Maf1 reduced pre-tRNA levels and prolonged lifespan, indicating that Maf1-mediated Pol III inhibition might be involved in the regulation of lifespan by mTOR pathway^15^. Moreover, *maf1*Δ cells showed a shorter lifespan under lower glucose conditions, and the short lifespan was rescued by introducing the plasmid encoding maf1 gene, which suggested that Maf1 was required for lifespan extension of *Schizosaccharomyces pombe*^31^. Maf1 was phosphorylated by TORC1 under high-calorie conditions, while it was dephosphorylated by PP2A and PP4 under calorie-restricted conditions. The phosphorylation status of Maf1 was associated with *S. pombe* lifespan and tRNA levels. Importantly, Maf1-dependent inhibition of tRNA transcription extended lifespan in fission yeast mainly by preventing genomic instability at tRNA genes, rather than inhibiting protein synthesis^31^. Further studies discovered that the break of tRNA genes was caused by replication-transcription conflicts, while Maf1 could limit Pol III-mediated transcription to maintain genomic integrity^32^. These findings indicate that transcription-related genomic instability may play an important role in the aging process. Intriguingly, Maf1 deletion increased the lifespan in worms and mice, which also indicated that Maf1 participated in lifespan regulation through complex mechanisms, not just by regulating Pol III output^33,34^.

### tRNA molecules in aging and lifespan

In addition, tRNA molecules are also involved in the regulation of aging and lifespan. Sagi et al.^35^ demonstrated that tRNA expression decreased with age in worms, and the higher *sup-7* tRNA levels at day 6 were associated with a longer lifespan. The decline in tRNA expression might cause protein misfolding, leading to the development of age-related diseases. Moreover, nuclear tRNA accumulation was related to the increased replicative lifespan in yeast^36^. In this context, deletion of tRNA exporter Los1 could significantly extend lifespan. Mechanistically, dietary restriction excluded Los1 from the nucleus in a manner dependent on Rad53 and mTOR, thereby promoting nuclear tRNA accumulation and transcription factor Gcn4 activation. Analogously, deletion of Nup100 facilitated the expression of Gcn4 by suppressing the nuclear export of tRNAs and thus contributed to the increased longevity in *S. cerevisiae*^16^. *nup100*Δ cells did not show tRNA splicing and aminoacylation defects, indicating that Nup100 was mainly responsible for the re-export of several mature tRNAs, such as tRNA^Tyr^, tRNA^Trp^, and tRNA^Ile^. Of note, the localization of Los1 and Msn5 (another protein involved in tRNA export) was not regulated by Nup100, which supported that Nup100 could regulate tRNA export in a manner distinct from them^16^. Together, the dysregulation of tRNA levels and transport may affect the lifespan of organisms.

### tRNA modifications in aging and lifespan

tRNAs always undergo a variety of post-transcriptional modifications, which affect tRNA stability, codon recognition, and even aminoacylation^37^. Strikingly, many studies have demonstrated that certain tRNA-modifying enzymes are involved in the regulation of cellular senescence and lifespan (Table 1). Alkylation repair homolog 8 (ALKBH8) is a tRNA methyltransferase involved in the formation of 5-methoxycarbonylmethyluridine (mcm^5^U), 5-methoxycarbonylmethyl-2′-O-methyluridine (mcm^5^Um), 5-methoxycarbonylmethyl-2-thiouridine (mcm^5^s^2^U), and 5-methoxycarbonylhydroxymethyluridine (mchm^5^U) at the anticodon wobble position of tRNAs^38–40^. ALKBH8-deficient mouse embryonic fibroblasts showed selenoprotein loss as well as a senescence phenotype characterized by increased levels of senescence-associated β-galactosidase (SA-β-Gal), heterochromatin foci, p16^INK4a^, and senescence associated secretory phenotype markers^41^. Another research found that dTrm7_34 and dTrm7_32, as functional orthologs of yeast TRM7 and human FtsJ RNA 2′-O-methyltransferase 1 (FTSJ1), catalyzed 2′-O-Methylation (Nm) at specific tRNAs in *Drosophila*^42^. Interestingly, Nm at position G_34_ limited the cleavage of tRNA^Phe^, while the 3′ terminal Cm_32_ might stabilize the tRNA^Phe^ fragments that were produced in dTrm7_34 mutants. Meanwhile, the mutant animals of dTrm7_34 and dTrm7_32 exhibited small RNA pathway dysfunctions, increased susceptibility to RNA virus infection, and shortened lifespan, suggesting that these two methyltransferases appeared to modulate the small RNA silencing and lifespan in adult flies^42^.

**Table 1:** Roles of tRNA-modifying enzymes in aging and lifespan.

tRNA-modifying enzymes	Subjects	Function	Effects	Mechanisms	References
ALKBH8	MEFs	Anti-aging	ALKBH8-deficient MEFs showed a senescence phenotype	Characterized by increased levels of SA-β-Gal, HCF, p16^INK4a^, and SASP markers	^41^
dTrm7_34 and dTrm7_32	*Drosophila*	Anti-aging	dTrm7_34 and dTrm7_32 mutant flies displayed reduced lifespan	–	^42^
mttu-1 and mtcu-2	*C. elegans*	Pro-aging	The lifespan of mtcu-2 and mttu-1 double mutants was significantly extended	Associated with OXPHOS dysfunction	^44^
Trm9	Yeast	Pro-aging	Trm9 deletion extended lifespan	Enhanced heat-shock resistance of mutants	^45^
hTRM9L	Cancer tissues	Pro-aging	Inhibited tumor growth via a senescence-like arrest	Related to SA-β-Gal activity and p21 expression	^46^
NSun2	Human fibroblasts	Anti-aging	Delayed the process of replicative senescence	Inhibited the translation of p27 by methylating p27 mRNA	^48^
NSun2	Human vascular endothelial cells	Pro-aging	Facilitated premature senescence induced by oxidative stress or high glucose	Promoted the translation of Shc adapter proteins by methylating Shc mRNA, thus activating p38MAPK and increasing ROS production	^49^
Dnmt2	*Drosophila*	Anti-aging	Dnmt2 was indispensable for maintaining the normal lifespan, and its overexpression prolonged lifespan	–	^52^
Dnmt2	Mouse fibroblasts	Anti-aging	Dnmt2 knockdown fibroblasts were susceptible to senescence under control conditions	Manifested by increased levels of p53 and p21, telomere shortening, oxidative stress and DNA damage	^53^
Dnmt2	Human fibroblasts	Anti-aging	Dnmt2 silencing induced cellular senescence	–	^54^

*tRNA* transfer RNA, *ALKBH8* alkylation repair homolog 8, *MEFs* mouse embryonic fibroblasts, *SA-β-Gal* senescence-associated β-galactosidase, *HCF* heterochromatin foci, *SASP* senescence-associated secretory phenotype, *OXPHOS* oxidative phosphorylation, *Trm9* tRNA methyltransferase 9, *hTRM9L* human Trm9-like protein, *NSun2* NOP2/Sun domain family member 2, *Shc* Src homology and collagen, *p38MAPK* p38 mitogen-activated protein kinase, *ROS* reactive oxygen species, *Dnmt2* DNA methyltransferase-2.

**Fig. 2:**
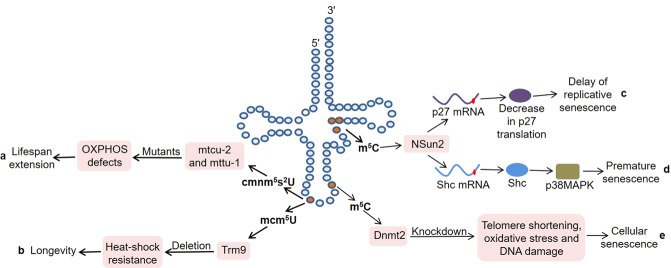
The dysregulation of tRNA-modifying enzymes in aging and lifespan. **a** The mtcu-2 and mttu-1 mutants show OXPHOS dysfunction, resulting in lifespan extension. **b** Trm9 deletion enhances heat-shock resistance of mutants, thereby supporting the link between longevity and cellular protection. **c** NSun2 inhibits the translation of p27 by methylating p27 mRNA, thereby delaying the process of replicative senescence. **d** NSun2 promotes the translation of Shc adapter proteins by methylating Shc mRNA. Subsequently, the increased Shc proteins activate p38MAPK, thereby facilitating premature senescence. **e** Dnmt2 knockdown fibroblasts are susceptible to senescence under control conditions, manifested by telomere shortening, oxidative stress, and DNA damage.

In mammals, mitochondrial translation optimization factor 1 (MTO1) catalyzed the formation of τm^5^U at anticodon position 34 in certain mitochondrial tRNAs (mt-tRNAs)^43^. The loss of MTO1 affected translation fidelity through defective tRNA modification in mice, resulting in tissue-specific oxidative phosphorylation (OXPHOS) defects. mttu-1 and mtcu-2 in *Caenorhabditis elegans* were the homologs of tRNA 5-methylaminomethyl-2-thiouridylate methyltransferase (TRMU) and MTO1^44^ (Fig. 2a). Notably, the lifespan of mttu-1 mutants was slightly extended at 20 °C, and that of mtcu-2 and mttu-1 double mutants was significantly extended, which was associated with the OXPHOS dysfunction in *C. elegans*. These findings indicate that these two modifying enzymes may be synergistic in regulating the lifespan of worms. But the underlying molecular mechanism requires further research. Fabrizio et al.^45^ discovered that the deletion of acyl-CoA binding protein (Acb1), tRNA methyltransferase 9 (Trm9) and CKA2 could significantly extend lifespan by performing a screen of a yeast homozygous deletion collection. Among them, Trm9 was responsible for the formation of 5-methoxycarbonyl-methyluridine (mcm^5^U) at position 34 in tRNA^Glu^ and tRNA^Arg3^. Importantly, their deletion enhanced the heat-shock resistance of mutants, thereby supporting the link between longevity and cellular protection (Fig. 2b). Furthermore, human Trm9-like protein (hTRM9L) was down-regulated in a variety of cancer tissues, and its re-expression significantly inhibited tumor growth in vivo^46^. hTRM9L induced a senescence-like phenotype related to SA-β-Gal activity and p21 expression. Meanwhile, hTRM9L could upregulate LIN9 and inhibit the hypoxic response, thereby exerting antitumor activity.

Mammalian NOP2/Sun domain family member 2 (NSun2) is responsible for the cytosine-5 methylation (m^5^C) in specific tRNA molecules, such as tRNA^Gly^, tRNA^Asp^, and tRNA^Val^^47^. Interestingly, NSun2 inhibited the translation of p27 by methylating the 5′-untranslated region (UTR) of p27 mRNA, thereby delaying the process of replicative senescence^48^ (Fig. 2c). At the same time, overexpression of the p27 5´UTR fragment could rescue the decrease of p27 and the increase of cyclin-dependent kinase 1 caused by NSun2 overexpression in 2BS cells, indicating that NSun2-mediated mRNA methylation played an important role in replicative senescence^48^. Cai et al.^49^ demonstrated that NSun2 promoted the translation of Src homology and collagen (Shc) adapter proteins, p66SHC, p52SHC, and p46SHC, by methylating Shc mRNA at multiple sites (Fig. 2d). Subsequently, the increased Shc proteins activated p38 mitogen-activated protein kinase (p38MAPK) and increased cellular reactive oxygen species production, thereby facilitating the premature senescence of human vascular endothelial cells induced by oxidative stress or high glucose.

In addition, DNA methyltransferase-2 (Dnmt2) specifically methylated cytosine 38 in the anticodon loops of tRNAs^50,51^. It has been confirmed that Dnmt2 is indispensable for maintaining the normal lifespan of *Drosophila*, and its overexpression can prolong lifespan^52^. Moreover, Dnmt2 was also related to the condition-dependent apoptosis and senescence in mouse fibroblasts^53^. On the one hand, Dnmt2 knockdown fibroblasts were more prone to apoptosis under the stimulation of hydrogen peroxide. On the other hand, these cells were more susceptible to senescence under control conditions, manifested by increased levels of p53 and p21, telomere shortening, oxidative stress, and DNA damage (Fig. 2e). Consistently, Dnmt2 silencing inhibited the proliferation of human fibroblasts and induced cellular senescence^54^. These findings indicate that Dnmt2 may serve as a novel regulator of longevity.

### tRNA aminoacylation in aging and lifespan

**Fig. 3:**
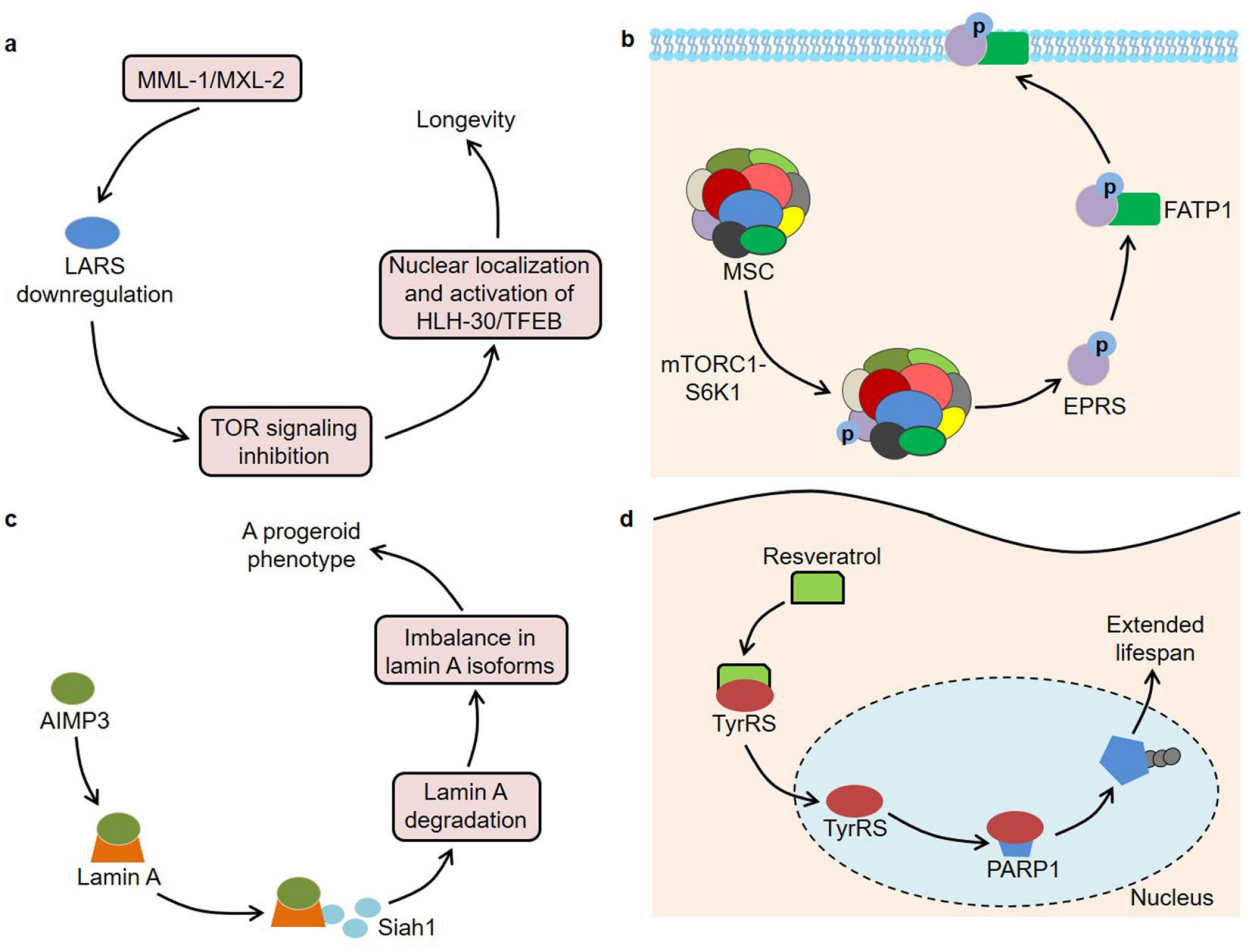
The dysregulation of ARSs in aging and lifespan. **a** MML-1/MXL-2 inhibits TOR activity by down-regulating LARS, leading to the nuclear localization and activation of HLH-30/TFEB. **b** mTORC1-S6K1 phosphorylates EPRS and thus induces its release from the MSC. The phosphorylated EPRS binds to FATP1 to promote its translocation to the plasma membrane. **c** AIMP3 interacts with lamin A and recruits Siah1, which leads to the degradation of lamin A as well as an imbalance in its isoform levels. **d** Resveratrol binds to TyrRS and facilitates its nuclear translocation. Then, TyrRS interacts with PARP1 and promotes its activation by stimulating NAD^+^-dependent auto-poly-ADP-ribosylation.

It is well known that tRNAs bind to their homologous amino acids through ARS-mediated aminoacylation, thereby transporting amino acids to the ribosome to participate in protein synthesis. In mammalian cells, one part of ARSs exists in free form, while the other part interacts with three ARS-interacting multi-functional proteins (AIMPs) to form a multi-tRNA synthetase complex (MSC)^55^. Intriguingly, ARSs and AIMPs are closely associated with aging and lifespan (Table 2). Previous studies found that a null mutation in mitochondrial leucyl-tRNA synthetase 2 (LARS2) was associated with longevity by screening 5690 genes of *C. elegans*^56^. The long-lived worms had impaired mitochondrial functions, manifested by lower ATP content and oxygen consumption. Furthermore, Mondo/Max-like complex (MML-1/MXL-2) played an important role in the lifespan extension induced by germline removal^57^ (Fig. 3a). In this context, MML-1/MXL-2 inhibited TOR activity by downregulating LARS, leading to the nuclear localization and activation of HLH-30/TFEB. Another research observed that prolyl-hydroxylase domain protein 1 (PHD1) levels were reduced in aging muscles, and PHD1 knockout mice had lower muscle mass^58^. PHD1 increased the stability of LARS by interacting with it, thereby ensuring leucine-mediated mTORC1 activation and maintaining muscle mass. These findings indicate that LARS participates in the biology of aging through different signaling pathways. Niehues et al. built a *Drosophila* model for Charcot–Marie–Tooth neuropathy by three mutations in glycyl-tRNA synthetase (GARS)^59^. Of note, the expression of these mutants, including GARS_E71G, GARS_G240R, and GARS_G526R, not only induced defects in neuronal morphology but also shortened the lifespan of flies in a dosage-dependent manner. In-depth research found that the mutant GARS proteins showed normal subcellular localization, but the overall protein synthesis in neurons was significantly reduced^59^. Interestingly, the heterozygous GARS^C201R^ mice had a normal lifespan, while this mutation significantly rescued the shortened lifespan caused by the SOD1^G93A^ mutation^60^. Therefore, more studies are still needed to explore the roles of GARS in lifespan regulation.

**Table 2:** Roles of ARSs in aging and lifespan.

ARSs	Subjects	Function	Effects	Mechanisms	References
LARS2	*C. elegans*	Pro-aging	A null mutation in LARS2 was associated with longevity	Associated with impaired mitochondrial functions, manifested by lower ATP content and oxygen consumption	^56^
LARS	*C. elegans*	Pro-aging	MML-1/MXL-2 promoted longevity	MML-1/MXL-2 inhibited TOR activity by down-regulating LARS, leading to the nuclear localization and activation of HLH-30/TFEB	^57^
LARS	Mouse	Anti-aging	PHD1 levels were reduced in aging muscles, and PHD1 knockout mice had lower muscle mass	PHD1 increased the stability of LARS, thereby ensuring leucine-mediated mTORC1 activation	^58^
GARS	*Drosophila*	Anti-aging	GARS mutations shortened the lifespan of flies in a dosage-dependent manner	Associated with impaired protein synthesis	^59^
Aats-met	*Drosophila*	Anti-aging	Aats-met mutations caused photoreceptor degeneration and reduced lifespan	Associated with increased ROS, oxidative phosphorylation defects and upregulation of mitochondrial unfolded protein response	^61^
MARS	*Drosophila*	Anti-aging	MARS inhibition shortened the lifespan of flies	Reduced the expression of AMPs genes	^62^
EPRS	Mouse	Pro-aging	Homozygous EPRS S999A mice exhibited low body weight, reduced adipose tissue mass and increased lifespan	mTORC1-S6K1 phosphorylated EPRS and induced its release from the MSC. Then, the EPRS bound to FATP1 to promote its translocation to the plasma membrane	^63^
SerRS	Cancer cells	Pro-aging	Induced cellular senescence	SerRS bound to telomere DNA repeats and enriched POT1 proteins to telomeres, leading to the shortening of telomeres	^64^
yars-2	*C. elegans*	Pro-aging	NMD-mediated RNA quality control was crucial for longevity	The down-regulation of yars-2, an NMD target, extended the lifespan of mutants	^65^
AIMP2	Mouse	Pro-aging	Contributed to the development of PD	Overexpression of AIMP2 activated PARP1, thereby resulting in an age-dependent dopaminergic neuronal loss	^67^
AIMP2-DX2	Cancer cells	Anti-aging	Blocked oncogene-induced apoptosis and senescence	Inhibited p14/ARF	^68^
AIMP3	Mouse	Pro-aging	AIMP3 levels were increased in aging human tissues, and AIMP3 transgenic mice had a premature aging phenotype	AIMP3 interacted with lamin A and recruited Siah1, which led to the degradation of lamin A and an imbalance in its isoform levels	^69^
AIMP3	hMSCs	Pro-aging	AIMP3 levels were increased, while the levels of miR-543 and miR-590-3p were decreased during the senescence of hMSCs	The two miRNAs inhibited the expression of AIMP3 by binding to AIMP3 transcripts	^70^
TyrRS	Mouse	Anti-aging	Mediated the lifespan extension regulated by resveratrol	Resveratrol bound to TyrRS and facilitated its nuclear translocation. Then, TyrRS interacted with PARP1 and promoted its activation	^94^
AIMP3	Mesenchymal stem cells	Pro-aging	AIMP3 down-regulation improved the age-related senescence of stem cells	HIF1α activated autophagy and inhibited mitochondrial respiration via suppressing the expression of AIMP3	^95^

*ARSs* aminoacyl-tRNA synthetases, *LARS2* leucyl-tRNA synthetase 2, *MML-1/MXL-2* Mondo/Max-like complex, *TOR* target of rapamycin, *PHD1* prolyl-hydroxylase domain protein 1, *GARS* glycyl-tRNA synthetase, *ROS* reactive oxygen species, *MARS* methionyl-tRNA synthetase, *AMPs* anti-microbial peptides, *EPRS* glutamyl-prolyl-tRNA synthetase, *S6K1* S6 kinase 1, *MSC* multi-tRNA synthetase complex, *FATP1* fatty acid transport protein 1, *SerRS* seryl-tRNA synthetase, *POT1* Protection of Telomeres 1, *NMD* nonsense-mediated mRNA decay, *AIMP2* ARS-interacting multi-functional protein 2, *PD* Parkinson’s disease, *PARP1* poly(ADP-ribose) polymerase-1, *AIMP2-DX2* AIMP2 lacking exon 2, *Siah1* seven in absentia homolog 1, *hMSCs* human mesenchymal stem cells, *TyrRS* tyrosyl-tRNA synthetase, *HIF1α* hypoxia-inducible factor 1α.

The mutations in Aats-met, a homolog of human methionyl-tRNA synthetase 2 (MARS2), caused photoreceptor degeneration and reduced lifespan of flies, which was associated with the increased ROS, oxidative phosphorylation defects and upregulation of mitochondrial unfolded protein response^61^. Moreover, inhibition of MARS could shorten the lifespan of flies by reducing the expression of anti-microbial peptides genes^62^. Arif et al. discovered that glutamyl-prolyl-tRNA synthetase (EPRS) was a downstream effector of the mTORC1 and p70 ribosomal protein S6 kinase 1 (S6K1) axis, which was involved in the biological processes of obesity and aging^63^ (Fig. 3b). In terms of mechanism, mTORC1-S6K1 phosphorylated EPRS at Ser^999^ and thus induced its release from the MSC. Subsequently, the phosphorylated EPRS bound to fatty acid transport protein 1 (FATP1) to promote its translocation to the plasma membrane and long-chain fatty acid uptake. Consistently, homozygous phospho-deficient EPRS S999A mice showed reduced adipose tissue, weight loss and longer lifespan, while replacement of the phospho-mimetic S999D allele restored the weight of s6k1-deficient mice to normal^63^. Furthermore, seryl-tRNA synthetase (SerRS) not only bound to telomere DNA repeats but also enriched Protection of Telomeres 1 (POT1) proteins to telomeres via direct interaction with POT1 in the nucleus^64^. The enrichment of POT1 led to the shortening of telomeres, thereby inhibiting the growth of HeLa cells by inducing cellular senescence. Intriguingly, the activity of nonsense-mediated mRNA decay (NMD) decreased with the age of *C. elegans*, and NMD could contribute to the longevity in *daf-2* mutant worms^65^. Further research has shown that downregulation of yars-2/tyrosyl-tRNA synthetase 2 (TyrRS2), an NMD target, effectively extends the lifespan of mutants, indicating that NMD-mediated RNA quality control plays an important role in organismal aging^65^.

Moreover, AIMPs, which mainly act as scaffolds in the MSC, is also associated with the aging process. It was reported that AIMP2 was a parkin substrate and contributed to the development of Parkinson’s disease (PD)^66^. Overexpression of AIMP2 could activate poly(ADP-ribose) polymerase-1 (PARP1), thereby resulting in an age-dependent dopaminergic neuronal loss in mice^67^. The PARP1 inhibitor AG014699 inhibited the degeneration of dopaminergic neurons in AIMP2 transgenic mice, indicating that PARP1 could be used as a target for PD treatment^67^. Notably, AIMP2-DX2, a splicing variant of AIMP2 lacking exon 2, was induced by oncogenes in human lung cancer cells and could block oncogene-induced apoptosis and senescence by inhibiting p14/ARF^68^. In addition, endogenous AIMP3 levels increased in aging human tissues, and AIMP3 transgenic mice had an obvious premature aging phenotype, which was manifested as earlier cessation of weight gain, hair loss, reduced bone mineral deposits in female and bone thickness, lordokyphosis, as well as wrinkled skin with reduced adipocytes^69^ (Fig. 3c). Mechanistically, AIMP3 interacted with lamin A and recruited seven in absentia homolog 1 (Siah1), which led to the degradation of lamin A. The lamin A degradation would result in an imbalance in its isoform levels, thus inducing organismal aging. Analogously, Lee et al.^70^ demonstrated that AIMP3 levels were increased, while the levels of miR-543 and miR-590-3p were decreased during the senescence of human mesenchymal stem cells. These two microRNAs (miRNAs) could inhibit the expression of AIMP3 by binding to AIMP3 transcripts, thereby delaying cellular aging.

### tRNA derivatives in aging and lifespan

Particularly, pre-tRNAs or mature tRNAs are cleaved into diverse subtypes of fragments under stress conditions, which are named tRNA derivatives^71^. Victoria et al. discovered that the circulating levels of 5′ tRNA halves derived from tRNA^Cys(GCA)^ and tRNA^Lys(CTT)^ were decreased, and those derived from tRNA^His(GTG)^ and tRNA^Asp(GTC)^ were increased with age in normal mice^72^. Importantly, the alterations in the levels of these 5′ tRNA halves were mitigated in the long-lived Ames dwarf mice. Likewise, another study found that the serum levels of certain specific 5′ tRNA halves changed significantly with age in mice, and their levels could be regulated by calorie restriction^73^. These results suggest that tRNA fragments may play vital roles in the anti-aging effects of dwarfism or calorie restriction. In some cases, RNA molecules harboring a 2′,3′-cyclic phosphate (cP-RNAs) at the 3′ end are generated from endoribonuclease-mediated RNA cleavage^74^. It was worth noting that cP-RNAs, mainly from tRNAs, rRNAs, and mRNAs, were abundantly present in mouse tissues, and their levels declined in an age-dependent manner^75^. Among them, the cP-RNAs derived from tRNAs were produced from the cleavage of anticodon loops and 3′-terminal CCA sequences. However, more studies are needed to explore the roles of tRNA derivatives in aging.

Of note, tRNA derivatives have been confirmed to be related to some age-related pathological processes, especially neurodegenerative diseases. Karaiskos et al.^76^ observed that the abundance of tRNA fragments in rat brains changed dynamically under the background of age. On the one hand, the levels of tRNA fragments derived from the 3′ end usually increased with age. On the other hand, the levels of tRNA fragments derived from the 5′ end were lower in the brains of middle-aged rats, while their levels were higher in the young and old rats. Interestingly, the potential targets of these fragments appeared to be enriched in neuronal functions and development, indicating that tRNA fragments might be involved in human aging and neurodegeneration^76^. Similarly, eight tRNA fragments were found to be differentially expressed in the brains of senescence-accelerated mouse prone 8 (SAMP8) mice, and these fragments seemed to regulate the brain function-associated pathways in a miRNA-like pattern^77^. For example, AS-tDR-011775 could act on myelin-associated oligodendrocyte basic protein or parkin (PARK2), thus contributing to the development of brain aging-associated diseases^77^. Conspicuously, cleavage and polyadenylation factor I subunit 1 (CLP1) could facilitate the efficient generation of tRNA exons by maintaining the integrity of the tRNA splicing endonuclease complex, and CLP1 kinase-dead mice showed progressive loss of lower motor neurons^78^. At the mechanistic level, loss of CLP1 activity led to the accumulation of 5′ leader exon tRNA fragments derived from pre-tRNA^Tyr^ (5′ Tyr-tRF) and p53-mediated cell death. Further research found that the 5′ Tyr-tRF promoted the p53-dependent neuronal cell death by interacting with pyruvate kinase M2 (PKM2)^79^. In addition, Balaskas et al. discovered that various tRNA and tRNA fragments were differentially expressed between young and old equine chondrocytes^80^. Importantly, certain 5′ tiRNAs, such as tiRNA His-GTG and tiRNA Glu-TTC, were induced in both old equine chondrocytes and high-grade osteoarthritis cartilage, indicating that tRNA fragments might be involved in the development of age-related cartilage diseases^80^.

## Conclusion and future perspective

Traditionally, tRNAs are considered as housekeeping molecules that mainly transport amino acids to the ribosome to participate in protein translation. After transcription, each tRNA needs to undergo a series of complex maturation processes to become functional^81^. In their metabolic process, defects in any step may cause various human diseases^82–84^. For example, the tRNA-modifying enzyme FTSJ1 was down-regulated in non-small cell lung cancer (NSCLC) tissues^85^. Importantly, FTSJ1 inhibited the growth of NSCLC cells by reducing the expression of DNA damage-regulated autophagy modulator 1. Furthermore, certain ARSs, including asparaginyl-tRNA synthetase, aspartyl-tRNA synthetase 2, and GARS, were associated with the development of neurological disorders^86–88^. As described above, tRNA-related metabolism, including tRNA transcription, tRNA molecules, tRNA modifications, tRNA aminoacylation, and tRNA derivatives, not only participates in cellular senescence but also plays a vital role in aging and longevity of organisms. In this context, studying tRNAs seems to provide new ideas for lifespan extension. However, the related molecular mechanism research is still in the initial stage, especially in the aspect of tRNA derivatives.

Indeed, some studies have begun to explore clinical transformations based on tRNA metabolism. Mutations in the human mitochondrial DNA (mtDNA) are implicated in age-associated disease phenotypes and aging^89,90^. Notably, specific mitoTALENs monomers for the tRNA^Ala^ m.5024C > T mutation could reduce the mutant mtDNA load and restore the tRNA^Ala^ levels in the muscle and heart of a mouse model of heteroplasmic mtDNA mutation^91^. It was reported that the natural phenol resveratrol contributed to extending the lifespan of organisms^92,93^. Further research showed that resveratrol could bind to the active site of TyrRS and facilitate its nuclear translocation^94^ (Fig. 3d). Then, TyrRS interacted with the C-domain of PARP1 and promoted its activation by stimulating NAD^+^-dependent auto-poly-ADP-ribosylation. Moreover, AIMP3 overexpression inhibited the functions of mesenchymal stem cells under hypoxic conditions, while the down-regulation of AIMP3 significantly improved the age-related senescence of stem cells^95^. Interestingly, hypoxia-inducible factor 1α (HIF1α) could activate autophagy and inhibit mitochondrial respiration via suppressing the expression of AIMP3, thereby delaying aging^95^. These findings provided a possible target for the regulation of aging. Another study found that the tRNA-derived fragments from the prefrontal cortex, cerebrospinal fluid and serum were differently expressed between PD patients and control samples, and they could distinguish PD from controls, indicating that tRNA fragments might serve as potential biomarkers for age-associated disease^96^. In conclusion, tRNA metabolism is closely related to aging and lifespan, and studying their relationship may become a hot topic in the future.
